# High Expression of PLAGL2 is Associated With Poor Prognosis in High-Grade Glioma

**DOI:** 10.3389/fgene.2021.787746

**Published:** 2022-02-09

**Authors:** Gang Wang, Wei Du, Lingyi Che, Xianzheng Gao, Ruihua Zhao, Juan Duan, Zhuoyu Gu, Qian Ma

**Affiliations:** ^1^ Department of Rehabilitation, First Affiliated Hospital, Zhengzhou University, Zhengzhou, China; ^2^ Department of Neurosurgery, First Affiliated Hospital, Zhengzhou University, Zhengzhou, China; ^3^ Genetic and Prenatal Diagnosis Center, Department of Gynecology and Obstetrics, First Affiliated Hospital, Zhengzhou University, Zhengzhou, China; ^4^ Department of Pathology, First Affiliated Hospital, Zhengzhou University, Zhengzhou, China; ^5^ Department of Medical Oncology, First Affiliated Hospital, Zhengzhou University, Zhengzhou, China; ^6^ Department of Cardiology, First Affiliated Hospital, Zhengzhou University, Zhengzhou, China; ^7^ Department of Thoracic Surgery, First Affiliated Hospital of Zhengzhou University, Zhengzhou, China

**Keywords:** PLAGL2, high grade glioma, prognosis, immunohistochemistry, TIMER

## Abstract

Pleomorphic adenoma gene like-2 (PLAGL2) has been implicated in the development and progression of diverse malignancies, including glioblastoma. An increasing number of studies have reported that dysregulated expression of PLAGL2 is a common phenomenon in different malignancies. However, the mechanism and biological functions of PLAGL2 in patients with high-grade glioma (HGG) remain unclear. In addition, the expression and clinical significance of PLAGL2 in HGG have not yet been reported. Herein, we investigated the expression patterns and prognostic values of PLAGL2 in patients with HGG by using various databases, including Tumor Immune Estimation Resource 2.0 (TIMER2.0), GENT2, ONCOMINE, GEPIA, Human Protein Atlas, and Gene Expression Omnibus datasets. In the present study, we analyzed the relationship between PLAGL2 mRNA expression and clinical parameters in 184 HGG cases and found that PLAGL2 presented positively high expression and was relevant to poor prognosis. Immunohistochemistry analysis confirmed the overexpression of PLAGL2 protein, which is mainly expressed in the nucleus of glioma. Additionally, a high level of expression of the PLAGL2 gene was associated with lower survival in progression-free survival and overall survival in GBM patients. The correlation analysis between PLAGL2 and immune infiltration related to the abundance of B cells, CD8^+^ T cells, CD4^+^ T cells, macrophages, DCs, and neutrophils was also performed using TIMER2.0. GSEA results showed that high PLAGL2 expression was associated with cell migration, proliferation, actin cytoskeletal, and angiogenesis. To sum up, our findings indicated that PLAGL2 could serve as an independent prognostic biomarker and might be a potential therapeutic target for HGG, which should be further investigated.

## Introduction

Malignant gliomas, encompassing a heterogeneous cluster of subtypes, are the most common primary central nervous system (CNS) tumors in both children and adults. Despite its low occurrence, glioma is considered one of the deadliest cancers worldwide, with the incidence at 5/100,000 each year (Dunn et al., 2012; Hu et al., 2016). According to the World Health Organization (WHO) criteria, gliomas are histologically categorized into grades I, II, III, and IV. While low-grade gliomas (LGGs) are often treated with surgery, radiotherapy, and chemotherapy, high-grade gliomas (HGGs) are aggressive and virtually incurable. An HGG, especially in its most aggressive form (glioblastoma), has an average survival of 1 year and often presents with progressive neurologic signs and symptoms characterized by radiochemotherapy resistance (Furnari et al., 2007; Taylor and Gerstner, 2013). Grade III astrocytoma and grade IV glioblastoma multiforme (GBM) are the most severe and incurable forms with dismal prognoses (Giese et al., 2003; Wen and Kesari, 2008). Thus, screening for new molecular biomarkers is crucial for improving prognosis and creating an individualized treatment for glioma.

A member of the PLAG gene family, PLAGL2, is a putative transcription factor that includes C2H2 zinc finger domains on the N-terminus with the DNA binding function. PLAGL2 was initially identified to have high structural conserved and similar DNA binding affinity with PLAG1 (Hensen et al., 2002; Ning et al., 2008). Aberrant PLAGL2 expression, found in leukemogenesis, participates in the development of acute myeloid leukemia (Landrette et al., 2005). Additionally, previous studies indicated that PLAGL2 was involved in the progression of various cancers, including colorectal cancer, lung adenocarcinoma, breast cancer, gastric cancer, and gastrointestinal cancer (Landrette et al., 2005; Hanks and Gauss, 2012; Xu et al., 2018; Li et al., 2019; Gao and Ye, 2020). A recent study also showed that PLAGL2 regulates the WNT/β-catenin pathway and contributes to the progression of GBM (Zheng et al., 2010). Interestingly, PLAGL2 possesses both carcinogenic and suppressive activities. Thus, although the expression and various fundamental carcinogenic processes of PLAGL2 have been extensively involved, the biological role of PLAGL2 in the development and progression of glioma remains largely unknown.

According to our knowledge, bioinformatics methods have not been used to investigate the role of PLAGL2 in GBM. In this study, we investigated the expression patterns and prognostic values of PLAGL2 in patients with HGG by using various databases, including TIMER2.0, GENT2, ONCOMINE, GEPIA, HPA, and GEO datasets.

## Materials and Methods

### Patients and Tissue Samples

A total of 184 surgically resected human glioma specimens were collected in the Department of Neurosurgery, the First Affiliated Hospital, Zhengzhou University, between 2011 and 2019. Among 184 patients, 115 (62.5%) were males and 69 (37.5%) were females. According to the WHO classification of CNS tumors (Wesseling and Capper, 2018), the collected neoplasms were classified as follows: 144 patients with grade III gliomas (including 2 astrocytoma, 109 anaplastic astrocytoma, 17 anaplastic oligoglioma, 4 oligoastrocytoma, 3 anaplastic oligoastrocytoma, and 9 anaplastic ependymoma) and 40 patients with GBM (grade IV). Collected clinical data included the gender, tumor location, histology, cell origin, tumor grade, clinical surgery status, and radiochemotherapy status. All tissues were snap-frozen in liquid nitrogen and stored at −80°C after resection. The clinicopathological features and surgery status of the patients are summarized in Table 1.

**TABLE 1:** Association of PLAGL2 expression in human glioma tissues with different clinicopathological features.

Variables	Group	PLAGL2 expression			
		N	Low	High	*p*-value
Gender	Female	69	29	40	0.094
	Male	115	63	52	
Location	Frontal lobe	67	35	32	0.673
	Parietal lobe	16	5	11	
	Occipital lobe	5	2	3	
	Temporal lobe	55	27	28	
	Insular lobe	2	1	1	
	Thalamus	10	6	4	
	Cerebellum	9	6	3	
	Brain stem	1	1	0	
	Lateral ventricles	10	4	6	
	Fourth ventricle	2	2	0	
	Other site	7	3	4	
Histology	Astrocytoma	2	0	2	0.22
	Anaplastic astrocytoma	109	59	50	
	Anaplastic oligoglioma	17	11	6	
	Oligoastrocytoma	4	2	2	
	Anaplastic oligoastrocytoma	3	1	2	
	Anaplastic ependymoma	9	5	4	
	Glioblastoma	40	14	26	
Cell origin	Astrocyte	151	73	78	0.596
	Oligodendroc	17	11	6	
	Oligodendroastrocyte	7	3	4	
	Ependymoma	9	5	4	
WHO grade	III	144	77	67	0.035
	IV	40	15	25	
Surgery	GTR	36	11	25	0.465
	NTR	43	10	33	
Radiochemotherapy	No	19	8	11	0.563
	Radiotherapy	84	49	35	
	Radiochemotherapy	81	46	35	

## Ethics Approval and Consent to Participate

The current study was approved by the Ethics Committees of the First Affiliated Hospital of Zhengzhou University before the clinical information was used for research purposes, and written informed consent was obtained from all participants. All study procedures were carried out in accordance with the ethical standards of the Helsinki Declaration.

### Immunohistochemistry

To block the endogenous peroxidase activity, the sections from 4% formalin-fixed and paraffin-embedded tissue blocks were dewaxed in xylene, rehydrated using graded concentration ethanol, and soaked in 0.3% hydrogen peroxide, which were cut into 5 μm-thick sections. Then, the tissue sections were rinsed in PBS. After having blocked in 10% goat serum (Gibco, Waltham, MA, USA) for nonspecific reactions, the sections were incubated with the anti-PLAGL2 polyclonal antibody (1:100, Abcam, Cambridge, MA, USA) overnight at 4°C. Negative control slides were processed in parallel with a nonspecific IgG. After washing, the sections were incubated with horse reddish peroxidase (HRP)-labeled anti-rabbit secondary antibody (Santa Cruz, CA, USA) for 1 h at room temperature. The slides were subjected to staining with 3, 30-diaminobenzidine solution and briefly counterstained with hematoxylin. Finally, the sections were rehydrated with xylene and ethanol, mounted in mounting resin, and examined with a microscope.

### Immunohistochemical Scoring

Two independent pathologists blinded to clinicopathological outcome calculated immunostaining reactions by multiplying the intensity and proportion of positive tumor cells. Briefly, the scores about the proportion of positive cells (0, <5%; 1, 6–50%; 2, >50%) and the scores about the staining intensity (0, no or weak staining; 1, moderate staining; and 2, strong staining) were combined. All the cases were divided into low expression and high expression subjected to the total score (median). Any discrepancy in the scoring was resolved by discussion between two pathologists in less than 10% of the examined slides, and an agreement was reached by rescoring the sections combined with a third pathologist in a multi-viewer microscope.

### Cancer Cell Line Encyclopedia

Cancer Cell Line Encyclopedia (CCLE; https://portals.broadinstitute.org/ccle) was downloaded from Gene Expression Omnibus (GEO, series GSE36133) and was used to identify the alternation of the expression of PLAGL2 across various cancer types. The Affymetrix Human Genome U133 Plus 2.0 DNA microarray gene expressions of 84 CNS cell lines were downloaded from CCLE in March 2012. Robust multiarray average (RMA) normalization and gene expression values was performed. One-way ANOVA was used to compare the expression of PLAGL2 in different types of cancer cell lines (Barretina et al., 2012; Cancer Cell Line Encyclopedia Consortium and Genomics of Drug Sensitivity in Cancer Consortium, 2015).

### ONCOMINE and GEO Analysis

The transcript level of PLAGL2 in glioma was ascertained by the ONCOMINE database (https://www.oncomine.org/), with a threshold set as such that *P* < 1E − 4, fold change >2, top gene rank 10% (Rhodes et al., 2004). The mRNA levels of PLAGL2 in cancer tissues were in contrast with that in normal tissues. Two one-sided T-tests were used to evaluate the differences. The cut-off of *p*-value was defined as 0.01, and the cut-off of fold change value was identified as 1. The microarray data of patients with glioma were downloaded from the GEO (https://www.ncbi.nlm.nih.gov/geo) public database under accession numbers GSE4290 and GSE53733 (Sun et al., 2006; Reifenberger et al., 2014).

### TCGA and CGGA Database

Normalized RNAseq expression data and corresponding clinical material were obtained from two independent databases: The Cancer Genome Atlas (TCGA) and Chinese Glioma Genome Atlas (CGGA) were both downloaded from Gliovis (http://gliovis.bioinfo.cnio.es/) (Bowman et al., 2017). TCGA is a public database (http://cancergenome.nih.gov/), which includes 29 cancer types, along with gene expression data and clinical information. Furthermore, a database (ID: mRNAseq 325) consisting of 325 glioma tissues with different grades (WHO I–IV) was downloaded from the CGGA (Wang et al., 2015; Liu et al., 2018). LGG and HGG were defined as WHO grades I–II and WHO grades III–IV according to the 2016 WHO classification of CNS tumors, respectively (Louis et al., 2021).

### UALCAN and GEPIA Analysis

The UALCAN (http://ualcan.path.uab.edu) and GEPIA datasets (http://gepia.cancer-pku.cn/) of TCGA gene expression were used to analyze the expression of PLAGL2 in GBM tissues and normal tissues (Asati et al., 2016). Two one-sided t-tests were used to evaluate the differences.

### Human Protein Atlas Dataset

PLAGL2 were analyzed at the protein and RNA levels in the Human Protein Atlas (HPA) dataset (https://www.proteinatlas.org/), which contained the immunohistochemistry staining images of 153 clinical GBM patients and their survival information with 54 females and 99 males (Pontén et al., 2008; Uhlen et al., 2017).

### TIMER Database Analysis

The Tumor Immune Estimation Resource (TIMER2.0) (http://timer.cistrome.org/) is a public web database for comprehensive assessments of abundance of tumor-infiltrating immune cells (TIICs) according to gene expression profiles (Li et al., 2020). We mainly performed PLAGL2 expression in GBM and the correlation between gene expression and abundance of immune infiltrates, involving B cells, CD8^+^ T cells, and CD4^+^ T cells, macrophages, neutrophils, dendritic cells (DCs), compared with purity in GBM and assessed how PLAGL2 is correlated with immune cell markers including CD8+T cells, T cells, B cells, monocytes, M1 macrophages, M2 macrophages, neutrophils, DCs, Th1 cells, type 2 helper T cells (Th2), Tfh cells, type 17 helper T cells (Th17), and Treg.

### Gent2 Database Analysis

GENT2 analysis (http://gent2.appex.kr/gent2/) revealed the gene expression in several types of cancer. The data from about 49,000 healthy and cancer individuals were analyzed in more than 30 different cancer types (Park et al., 2019).

### GeneMANIA and STRING Web Analysis

The GeneMANIA (http://www.genemania.org/) web database revealed the interaction relationship of a gene and the performed gene function, physical interaction, pathway, co-expression, and localization (Warde-Farley et al., 2010). The STRING database (https://string-db.org/) was utilized to obtain the interaction network of related genes for PLAGL2, including direct (physical) and indirect (functional) associations. Thus, nodes represent genes, whereas links conduct connected networks (Szklarczyk et al., 2017; Szklarczyk et al., 2019).

### Gene Set Enrichment Analysis

To investigate the potential mechanisms underlying the interaction of PLAGL2 expression on glioma progression, a GSEA was conducted to screen out whether some biological pathways showed statistically significant differences between high and low PLAGL2 expression groups (Subramanian et al., 2005). Pre-defined gene sets were obtained from the Molecular Signatures database, MSigDB (http://software.broadinstitute.org/gsea/msigdb) (Subramanian et al., 2005). Gene sets: M457 (CYTOSKELETON), M2001 (WU_CELL_ MIGRATION), and M16210 (CELL_PROLIFERATION_GO_0008283), M14493 (Angiogenesis_GO_ 0001525), M39729 (WP_VEGFAVEGFR2_ SIGNALING_PATHWAY) (Liberzon et al., 2011). Normalized enrichment score (NES) and false discovery rate (FDR) were calculated to verify the significant difference for GSEA. For each analysis, gene set permutations were implemented for 1,000 times. Gene sets with an FDR <0.05 and normal *p* <0.05 were viewed as significantly enriched.

### Statistical Analysis

Statistical analyses were performed by SPSS 22.0 software, and the results were presented as the mean ± standard deviation (SD). Student’s t-test was performed to detect significant differences between groups. The relationships of PLAGL2 expression and various clinicopathological features were estimated by chi-squared test. Analyses with more than two groups were performed by the one-way ANOVA test. The Kaplan–Meier with log-rank test was used to analyze the survival data. The correlation analysis between PLAGL2 expression and immune-related markers was performed using Pearson correlation. Univariate and multivariate Cox regression analyses were used to analyze the clinical prognostic parameters and related independent risk factors. The results were considered significant at *p < 0.05, **p < 0.01, and ***p < 0.001.

## Results

### mRNA Expression Value of PLAGL2 in Different Cancer Types

**FIGURE 1:**
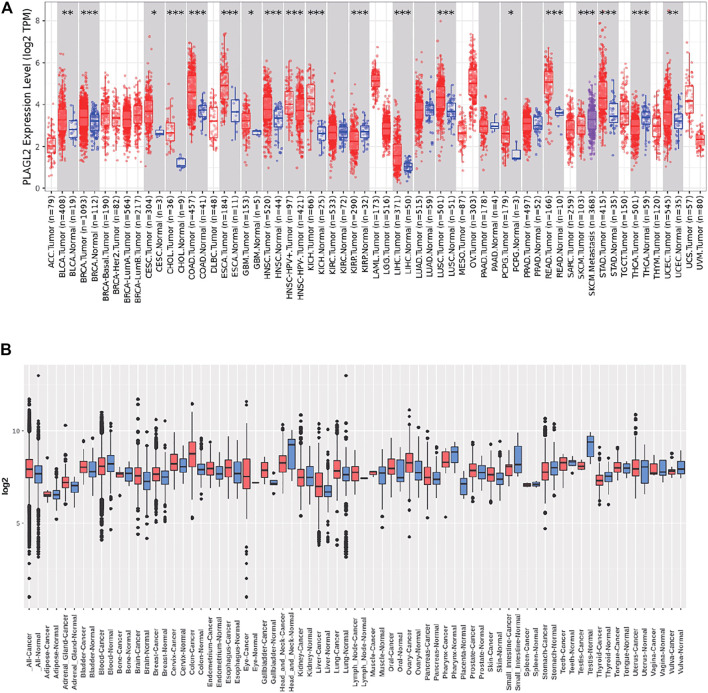
Tissue-wide mRNA expression profile of PLAGL2 across different types of cancer using the TIMER and GENT2 databases. **(A)** Expression of PLAGL2 in different types of cancers compared with healthy tissues in data derived from TIMER2.0 database. **(B)** PLAGL2 mRNA expression pattern in various tumor types was obtained from the GENT2 database. Red boxplot specifies various cancer samples, whereas blue boxplot designates normal samples. *p < 0.05, **p < 0.01, **p < 0.001.

We performed pan-cancer analysis derived from TCGA by adopting Timer 2.0 and Gent2 so as to estimate the expression pattern of PLAGL2. According to Timer analysis, PLAGL2 was upregulated in bladder, breast, cervix, bile ducts, colorectal, esophagus, kidney, liver, lung, stomach, and CNS cancer compared with normal cases (Figure 1A, Supplementary Table S1), whereas it was downregulated in renal and thyroid cancers.

Moreover, we estimated the PLAGL2 expression data profiles of 72 paired cancers vs. normal tissues by utilizing the HG-U133 microarray (GPL570 platform) of the Gent2 database. The results showed that PLAGL2 was upregulated in several cancers, including adrenal gland, brain, breast, cervix, colon, esophagus, liver, lung, oral, ovary, and skin. Conversely, the upregulation of PLAGL2 was reported in the pharynx, small intestine, testes, stomach, head, and neck (Figure 1B, Supplementary Table S2). Combining the above two results, systematic mRNA expression analysis with the Timer and GENT2 databases have shown that PLAGL2 was consistently upregulated across a wide range of cancer types that included breast, cervix, colon, esophagus, liver, lung, and brain.

Next, we used these respective cancer types in which significant upregulation was found for further investigation. In addition, we selected CCLE and HPA databases to further validate the PLAGL2 expression in the cell line. The CCLE analysis results indicated a certain degree of PLAGL2 expression in 84 CNS cell lines containing glioma cancer cell lines (Supplementary Table S3). In GBM U251-MG cells from HPA, the immunofluorescent staining of human cell line U251-MG revealed that PLAGL2 was localized to the nucleoplasm and cytosol, as shown in Supplementary Figure S1, where green represents antibody, red means microtubules, and blue means nucleus.

### PLAGL2 Expression Was Upregulated in HGG

**FIGURE 2:**
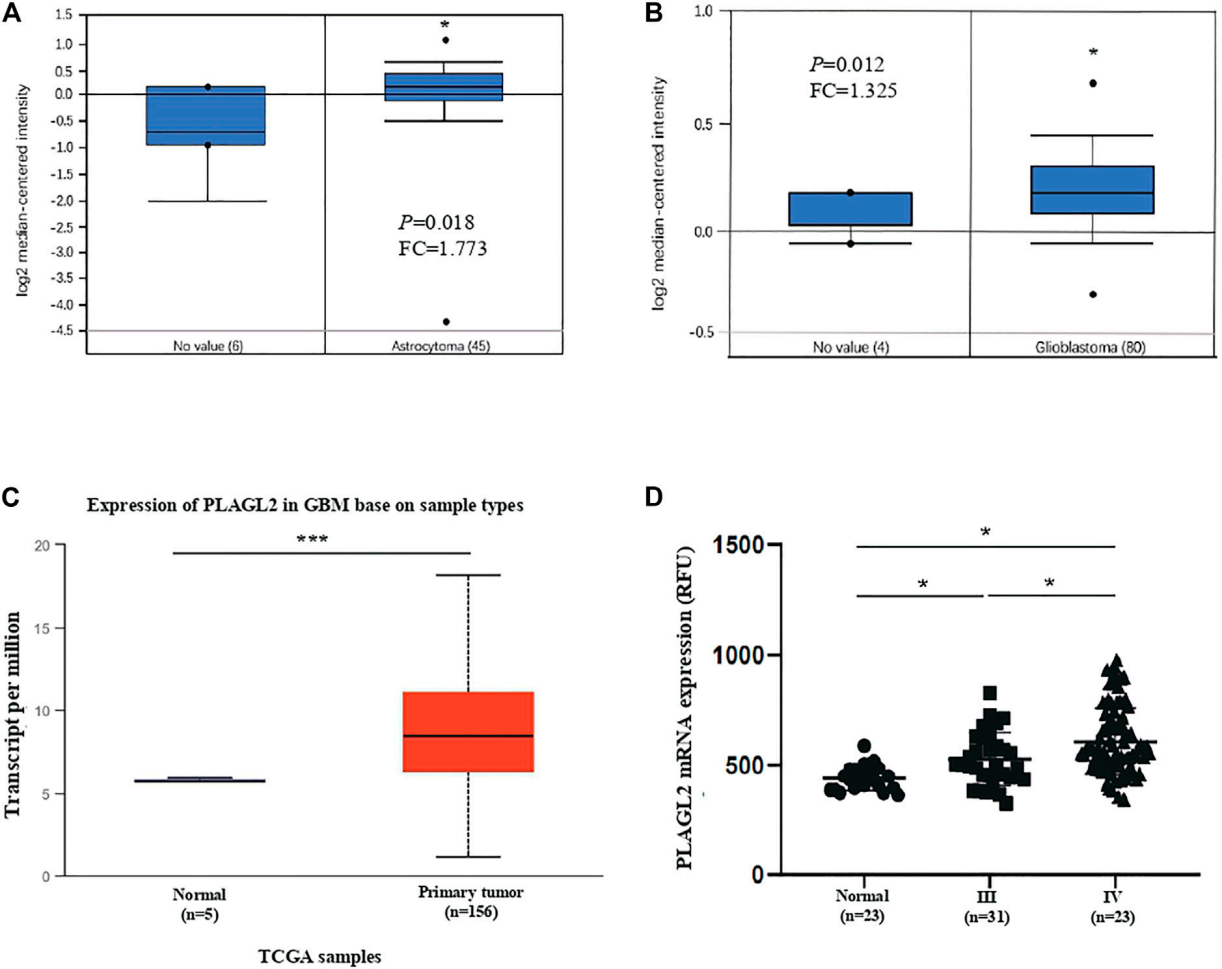
Analysis of PLAGL2 expression in HGG and normal brain tissues with microarray datasets of glioma and log2 median intensity mRNA. **(A,B)** PLAGL2 mRNA expression in human gliomas was analyzed in GBM tissues and non-tumor brain tissues from the Richman brain and Murat brain datasets in ONCOMINE database. **(C)** Boxplot showing expression of PLAGL2 increased in GBM cancer tissues compared with adjacent normal in UALCAN. **(D)** Expression of PLAGL2 in glioma samples of the GSE4290 dataset.

Firstly, we assessed any deregulated differences of the PLAGL2 mRNA level in glioma compared to normal brain tissues, which were tested using the data in human glioma from publicly available microarray datasets (https://www.oncomine.org/) (Rhodes et al., 2004). Based on the ONCOMINE database, PLAGL2 has significantly high expression in glioma tissues (astrocytoma and glioblastoma) as compared with normal tissues (Figures 2A,B) (Rickman et al., 2001; Murat et al., 2008). Similarly, as shown in Figure 2C and Supplementary Figures S2A,B, PLAGL2 are upregulated in GBM from the TCGA sample based on UALCAN and GEPIA databases. Meanwhile, the results in LGG indicated that patients with glioma also had higher expression levels of PLAGL2 compared with non-tumor tissue, which indicated that PLAGL2 expression was closely linked to the malignancy of glioma (Supplementary Figure S2C). To further characterize the relationship of PLAGL2 expression level and tumor specimens, we explored the expression levels of PLAGL2 in the GEO database (GSE4290). According to the WHO grade, the results showed that the expression of PLAGL2 mRNA was greatly higher in grades III and IV than in normal brain tissues (Figure 2D). Moreover, GENT2 database analysis showed that PLAGL2 expression increases with tumor grade (Supplementary Figure S3).

IDH mutation and 1p/19q co-deletion status are well-known clinically relevant molecular markers in glioma. Herein, we analyzed the correlation between the mRNA levels of PLAGL2 and major clinical features of GBM patients in CGGA, including grade level, IDH mutation status, 1p/19q co-deletion status, gender, and age. As shown in Supplementary Figures S4A−E, PLAGL2 were differently expressed in different grade groups and significantly associated with IDH mutation, 1p/19q co-deletion, gender, and age status, respectively. Overall, the above results illustrated that the mRNA level of PLAGL2 was significantly associated with clinical and pathological features, such as grade, gender, age, IDH mutation, and 1p/19q co-deletion status, which were also considered as potential prognostic and diagnostic predictors of HGG with poorer treatment.

### Protein Levels of PLAGL2 in Patients With GBM and its Association With Clinicopathological Characteristics

**FIGURE 3:**
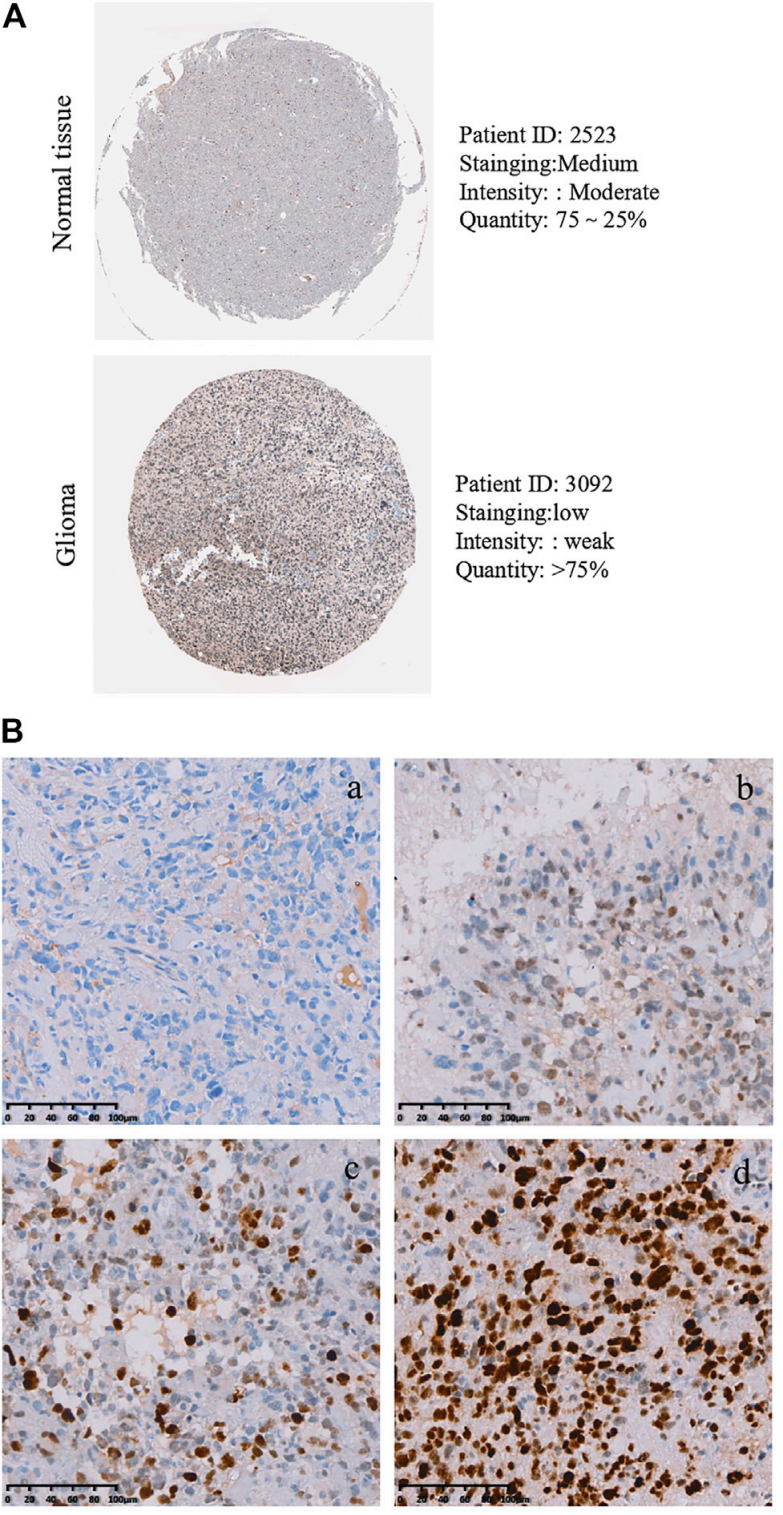
Protein levels of PLAGL2 protein expression in HGG samples. **(A)** Valiation of PLAGL2 in HGG from HPA. **(B)** Representative immunohistochemistry images of negative (WHO III), low (WHO III), moderate (WHO IV) and high (WHO IV) PLAGL2 protein expression (original magnification ×200).

As described above, the mRNA levels of PLAGL2 were upregulated in GBM, so we hypothesized that the protein levels of the PLAGL2 were also elevated. In the HPA dataset, the IHC staining data revealed that PLAGL2 was mainly localized in the nucleus and had higher expressions in GBM tissues (Figure 3A). To validate the above findings and investigate the clinicopathological roles and distribution of PLAGL2 expression in HGG, an immunohistochemical analysis of the 184 paraffin-embedded HGG tissue blocks was performed. These gliomas comprised 144 grade III and 40 grade IV tumors. Representative immune-histochemical staining of PLAGL2 in gliomas is illustrated in Figure 3B (a-d). PLAGL2 expression levels were significantly lower in grade III (a-b) compared to grade IV gliomas (c-d). A strong expression of PLAGL2 was observed in the nuclei of tumor cells. Additionally, there was no positive signal in any non-neoplastic tissues. The cases were divided into high or low PLAGL2 expression group by the median of PLAGL2 immunostaining score in cancer tissues (n = 92 for each group). As listed in Table 1, the expression of PLAGL2 was significantly correlated with WHO grade (*p* < 0.05), but not with gender, tumor locus, histology, cell origin, treatment regimen, and radiochemotherapy. Meanwhile, considering the GBM group, the analysis results showed that the expression of PLAGL2 was significantly correlated with gender (*p* < 0.01), but not with tumor locus and radiochemotherapy (Table 2). Those results suggested that PLAGL2 expression was more prevalent in aggressive gliomas.

**TABLE 2:** Association of PLAGL2 expression in GBM tissues with different clinicopathological features.

Variables	Group	PLAGL2 expression			
		N	Low	High	*p*-value
Gender	Female	15	5	10	<0.01
	Male	25	9	16	
Location	Frontal lobe	22	8	14	0.99
	Temporal lobe	16	6	10	
	Insular lobe	3	1	2	
Histology	Glioblastoma	40	14	26	
Cell origin	Astrocyte	40	12	18	
Radiochemotherapy	No	2	1	1	0.85
	Radiotherapy	19	7	12	
	Radiochemotherapy	19	6	13	

### High PLAGL2 Expression Predicted Poor Prognosis and Prognostic Value in HGG Patients

**FIGURE 4:**
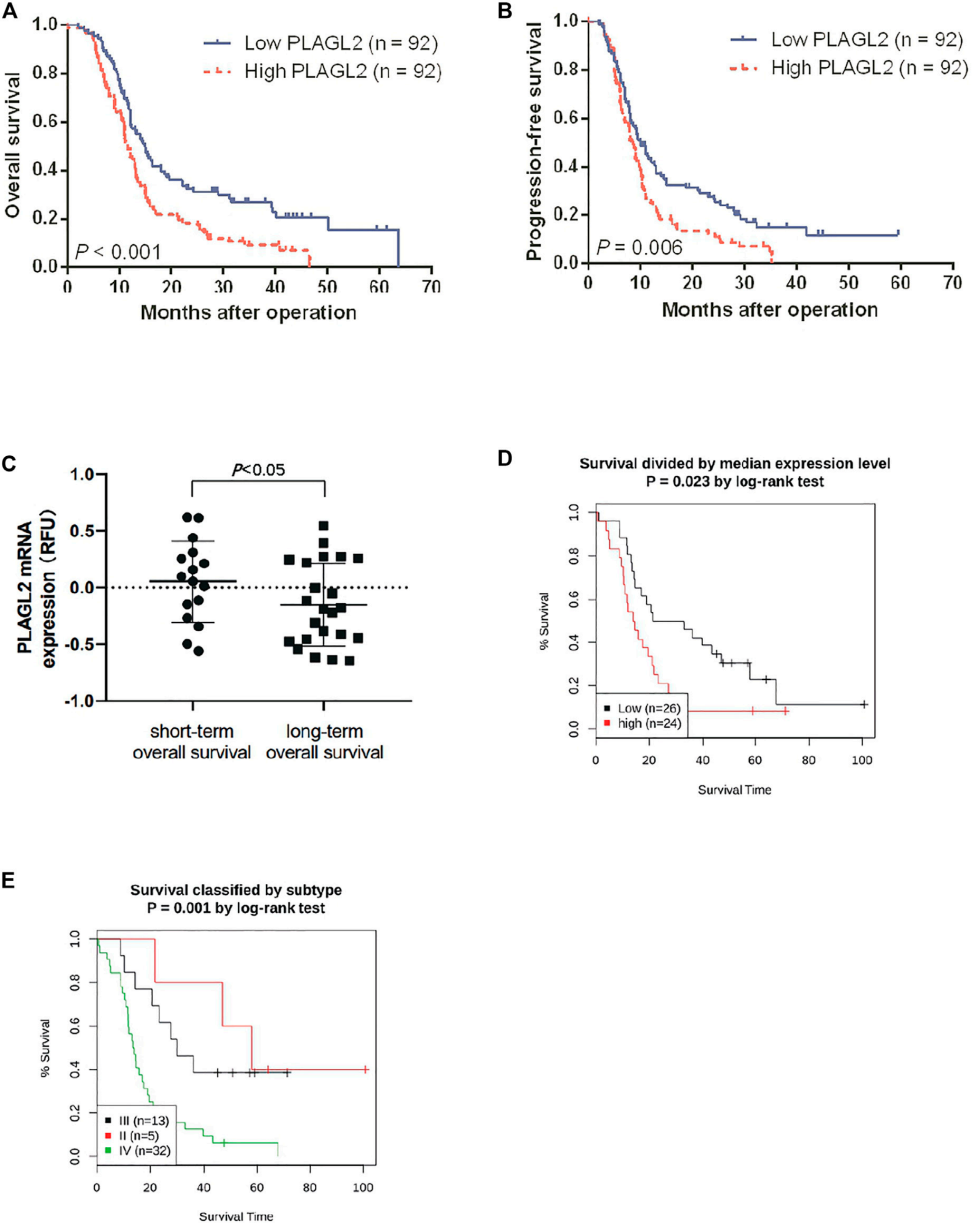
Association between PLAGL2 expression and OS and PFS of HGG patients assessed by Kaplan–Meier survival curves and GENT2 database. **(A,B)** Patients with high expression had significantly shorter OS and PFS time than those with low expression, *p* < 0.001 and *p* < 0.01, respectively. **(C)** High PLAGL2 expression is associated with shorter OS time in GSE53733 (*p* < 0.05). **(D)** Survival divided by PLAGL2 median expression level (log-rank test, *p* = 0.023) **(E)** Survival classified by subtype (log-rank test, *p* = 0.001).

Survival length was determined from the time of primary tumor surgery to the time of death or the last follow-up. To investigate the effect of PLAGL2 overexpression on the overall survival (OS) and progression-free survival (PFS) of HGG patients and explore the prognostic value of PLAGL2 in HGG, survival analysis was performed based on information of 184 HGG patients. As shown in Figures 4A,B, HGG patients with high PLAGL2 expression showed significantly worse OS and PFS than those with low PLAGL2 expression (*p* < 0.001 and *p* = 0.006, respectively). Similarly, we explored the expression levels of PLAGL2 in the GEO database. GSE53733 experiments indicated that the expression of PLAGL2 in the short-term OS group was significantly higher than that in the long-term OS group (Figure 4C). Moreover, we used the Gent2 database to analyze the survival plot or KM plot for PLAGL2 expression against HGG (Figure 4D). The GENT2 database was used to obtain the log-rank test curve, which showed that high levels of the PLAGL2 expression group had a lower survival rate in HGG patients (*p* < 0.05). Moreover, a low prognostic value was observed in high-grade stage groups (*p* = 0.001) (Figure 4E). Next, HGG patients with primary glioma and recurrent glioma in CGGA were grouped into cohorts based on the median expression levels of PLAGL2 expression. Similarly, we found that those with low expression had a better prognosis compared to those with higher expression levels in primary and recurrent glioma (Supplementary Figures S5A, 5B). A higher expression level of PLAGL2 also predicted a poor prognosis for WHO grade III and IV glioma patients compared to those with lower expression (Supplementary Figures S5C−F). Furthermore, the AUC values of the prognostic model for the 1-, 2-, and 3-year survival rate prediction in the TCGA cohort were 0.708, 0.715, and 0.713, respectively (Supplementary Figure S6A). The AUC values for disease specific survival in the TCGA cohort at 1, 2, and 3 years were 0.713, 0.72, and 0.714, respectively (Supplementary Figure S6B). These findings confirmed that the prediction performance of the prognostic signature was considerably promising and the survival analysis accurately predicted the prognosis of patients with GBM and LGG. An ROC curve analysis was also performed in that the high sensitivity and specificity of differentiating IDH status, histological, and 1p19q co-deletion status are based on the OS of the TCGA database (Supplementary Figure S7).

To further determine the prognostic value of PLAGL2 expression, we performed the univariate and multivariate Cox regression analyses based on OS and PFS of HGG patients. As shown in Tables 3, 4, PLAGL2 expression, age class, and WHO grade resulted as independent prognostic factors for the OS and PFS of HGG patients. Next, the results of univariate and multivariate Cox regression analyses suggested that clinical histology, age, and 1p19q co-deletion status were associated with the OS of HGG patients **(** Supplementary Table S4 **)**.

**TABLE 3:** Univariate and multivariate Cox proportional hazards analysis of OS in HGG cohort.

Variables	Univariate analysis		Multivariate analysis	
	HR (95%CI)	*p*-value	HR (95%CI)	*p*-value
PLAGL2 expression	1.707 (1.238–2.353)	**0.01**	1.540 (1.114–2.130)	**0.009**
Age	1.981 (1.419–2.764)	**<0.001**	1.874 (1.341–2.618)	**<0.001**
WHO	2.364 (1.626–3.439)	**<0.001**	2.118 (1.451–3.093)	**<0.001**
Gender	0.889 (0.643–1.231)	0.481		
Surgery	0.944 (0.556–1.602)	0.831		
Radiochemotherapy	0.921 (0.676–1.255)	0.605		

**TABLE 4:** Univariate and multivariate Cox proportional hazards analysis of PFS in HGG cohort.

Variables	Univariate analysis		Multivariate analysis	
	HR (95%CI)	*p*-value	HR (95%CI)	*p*-value
PLAGL2 expression	1.543 (1.124–2.119)	**0.007**	1.449 (1.053–1.994)	**0.023**
Age	1.958 (1.408–2.722)	**<0.001**	1.927 (1.383–2.685)	**<0.001**
WHO	3.078 (2.097–4.518)	**<0.001**	3.037 (2.056–4.488)	**<0.001**
Gender	0.921 (0.669–1.267)	0.615		
Surgery	0.888 (0.525–1.502)	0.658		
Radiochemotherapy	0.842 (0.627–1.131)	0.253		

### Correlation Between PLAGL2 mRNA Expression and Immune Infiltration Cells by TIMER

**FIGURE 5:**
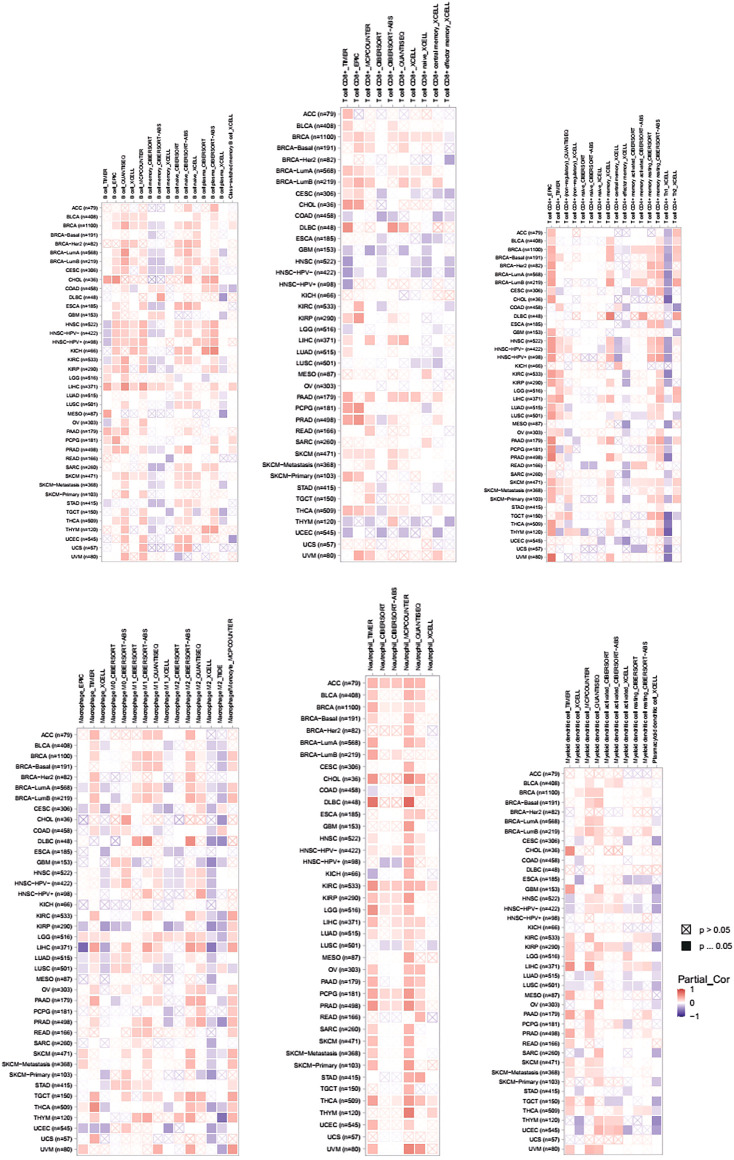
Associations of PLAGL2 expression to tumor purity and immune infiltration in various cancers. Association heatmap of immune cell infiltration based on B cells, CD8^+^ T cells, CD4^+^ T cells, macrophages, neutrophils, and DCs in multiple cancer types.

**FIGURE 6:**
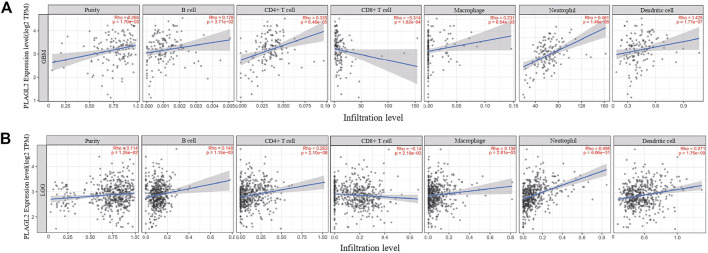
Association analysis between mRNA levels of PLAGL2 and tumor immune infiltration in GBM and LGG. **(A,B)** PLAGL2 expression in GBM tissues was positively associated with tumor purity, B cells, CD4^+^T cells, macrophages, neutrophils, and DCs, compared with negatively related to CD8^+^T cells.

Tumor-infiltrating lymphocytes (TILs) could affect a patient’s OS and regulate the tumor response to therapies (Park et al., 2020). By combining the above results, we found that PLAGL2 was significantly upregulated and associated with OS and PFS in HGG patients. Cumulative studies have suggested that PLAGL2 expression has a vital role in the malignancy of various cancers (Zheng et al., 2010; Zhao et al., 2020; Wu et al., 2021). To explore the pan-cancer correlation between PLAGL2 expression and immune infiltration, we first evaluated the abundance of immune cell infiltration. As shown in Figure 5, we adopted the TIMER database to illustrate the profiles of PLAGL2 correlating with various immune infiltrations, which showed that it was positively correlated with the immune cell infiltration levels of neutrophils. However, data indicated that it was obviously contradicted in B cells, macrophages, CD4^+^T cells, CD8^+^T cells, and DCs. Next, we then performed a correlation analysis between PLAGL2 and tumor-infiltrating immune cells, including B cells, CD4^+^T cells, CD8^+^T cells, macrophages, neutrophils, and DCs by the TIMER database in GBM patients to evaluate the immunotherapy effect. Tumor purity acted as a critical factor, which affected the analysis of immune infiltration in the analysis of the genomic approach. As shown in Figure 6A, PLAGL2 was positively associated with purity (rho = 0.265, *p* = 0.0017), B cells (rho = 0.178, *p* = 0.0371), CD4^+^T cells (rho = 0.335, *p* < 0.001), macrophages (rho = 0.231, *p* < 0.01), neutrophils (rho = 0.461, *p* < 0.001) and DCs (rho = 0.428, *p* < 0.001), and negatively associated with CD8^+^T cells (rho = -0.314, *p* < 0.001) in GBM. Additionally, we also investigated the immune infiltration status of PLAGL2 related to immune cells in LGG. Similarly, the purity (rho = 0.265, *p* = 0.0125), B cells (rho = 0.148, *p* = 0.00115), CD4^+^T cells (rho = 0.253, *p* < 0.001), CD8^+^T cells (rho = -0.14, *p* = 0.00218), macrophages (rho = 0.136, *p* = 0.00281), neutrophils (rho = 0.495, *p* < 0.001), and DCs (rho = 0.271, *p* < 0.001) were significantly correlated with PLAGL2 in LGG (Figure 6B). In general, our findings revealed distant associations between PLAGL2 expression with cancer purity and immune cells infiltration, such as B cells, CD8^+^T cells, CD4^+^T cells, and macrophages and neutrophils, while DCs revealed that PLAGL2 had a vital role in immune infiltration in GBM, which modulated the infiltration of immune cells to tumor tissues.

### The Levels of PLAGL2 Expression Correlated With Gene Markers of Different Cohorts of Immune Cells in GBM

Next, we comprehensively clarified the correlation of PLAGL2 and the status of infiltrating immune cells in GBM based on a cohort of immunological makers using the TIMER2.0 database. The gene markers of immune cells were used to analyze and identify the immune cells, including CD8^+^ T cells, CD4^+^ T cells, regulatory T cells, B cells, tumor-associated macrophages (TAMs), M1 and M2 macrophages, monocytes, neutrophils, DCs, and natural killer (NK) cells. Meanwhile, NK cells, T helper 1 (Th1), T helper 2 (Th2), follicular helper T (Tfh), T helper 17 (Th17), and regulatory T (Tregs) were also analyzed. The correlation analysis data were adjusted for tumor purity. An analysis of the TIMER database showed that PLAGL2 expression in GBM was significantly correlated with the expression of marker genes in CD8^+^T cells, general T cells, B cells, monocytes, M1 and M2 macrophages, neutrophils, DCs, NK cells, Th1, Th2, Tfh, Th17, and Treg (Table 5). Interestingly, PLAGL2 expression was critically correlated with the expression of markers of specific immune cells such as CD8^+^T cell marker, CD8B (r = -0.314, *p* < 0.001); T-cell marker, CD3D (r = -0.284, *p* < 0.001) and CD2 (r = -0.185, *p* < 0.05); B-cell marker, CD79B (r = -0.178, *p* < 0.05); monocyte marker, CD14 (r = 0.171, *p* < 0.05) and CD115 (r = 0.175, *p* < 0.05); M1 macrophage marker, CCL5 (r = -0.184, *p* < 0.05), IL18 (r = -0.266, *p* < 0.01) and COX (r = 0.396, *p* < 0.001); M2 macrophage marker, CD163 (r = 0.178, *p* < 0.05), neutrophil marker, CD11b (r = 0.337, *p* < 0.001); DC marker, HLA-DQB1 (r = 0.221, *p* < 0.01) and BDCA4 (r = 0.374, *p* < 0.001), and NK cell marker, NCR3 (r = -0.219, *p* < 0.01).

**TABLE 5:** Correlation analysis between PLAGL2 and related genes of immune cells in TIMER database.

Description	Gene markers	GBM (n = 158)			
		None		Purity	
		rho	*P*	Rho	*P*
CD8^+^ T cell	CD8A	−0.1	0.247	−0.188	*
	CD8B	−0.314	***	−0.271	**
T cell	CD3D	−0.284	***	−0.43	***
	CD2	−0.185	*	−0.402	***
B cell	CD79A	−0.035	0.682	−0.051	0.55
	CD79B	−0.178	*	−0.188	*
Monocyte	CD14	0.171	*	−0.563	***
	CD115(CSF1R)	0.175	*	−0.515	***
M1 Macrophage	CCL5	−0.184	*	−0.469	***
	IL18	−0.266	**	−0.608	***
	COX (PTGS2)	0.396	***	−0.331	***
M2 Macrophage	CD163	0.178	*	−0.466	***
	VSIG4	−0.055	0.525	−0.598	***
Neutrophils	CD11b (ITGAM)	0.337	***	−0.552	***
	CCR7	−0.033	0.706	−0.308	***
Dendritic cell	HLA-DQB1	0.221	**	−0.355	***
	BDCA-4(NRP1)	0.374	***	−0.2	*
Natural killer cell	NCR3	−0.219	**	−0.206	***
Th1	TBX2	0.407	***	0.228	**
	STAT4	−0.016	0.854	−0.352	***
Th2	CD14	0.171	*	−0.563	***
	GATA3	0.177	*	0.011	0.896
	STAT6	0.265	**	−0.429	***
	STAT5A	0.43	***	−0.277	***
Tfh	BCL6	0.444	***	0.001	0.993
Th17	STAT3	0.608	***	0.046	0.589
Treg	FOXP3	0.231	**	−0.165	0.0534
	STAT5B	0.612	***	0.292	***
	TGFB (TGFB1)	0.363	***	−0.361	***

Moreover, the expression of PLAGL2 was associated with the expression of markers of specific subsets of T cells in GBM, which included the Th1 marker, TBX2 (r = 0.407, *p* < 0.001); Th2 marker, CD14 (r = 0.171, *p* < 0.05), GATA3 (r = 0.177, *p* < 0.05), STAT6 (r = 0.265, *p* < 0.01), STAT5A (r = 0.43; *p* < 0.001); Tfh marker, BCL6 (r = 0.444, *p* < 0.001); Th17 marker, STAT3 (r = 0.608, *p* < 0.001); Treg marker, FOXP3 (r = 0.231, *p* < 0.01), STAT5B (r = 0.612, *p* < 0.001), and TGFB (r = 0.363, *p* < 0.001). However, PLAGL2 expression did not reveal any significant correlation with CD4^+^ T cells and TAMs. In summary, a comprehensive analysis indicated that PLAGL2 expression was strongly correlated with infiltration of immune cells in GBM.

### Interacting Network Analysis for PLAGL2

**FIGURE 7:**
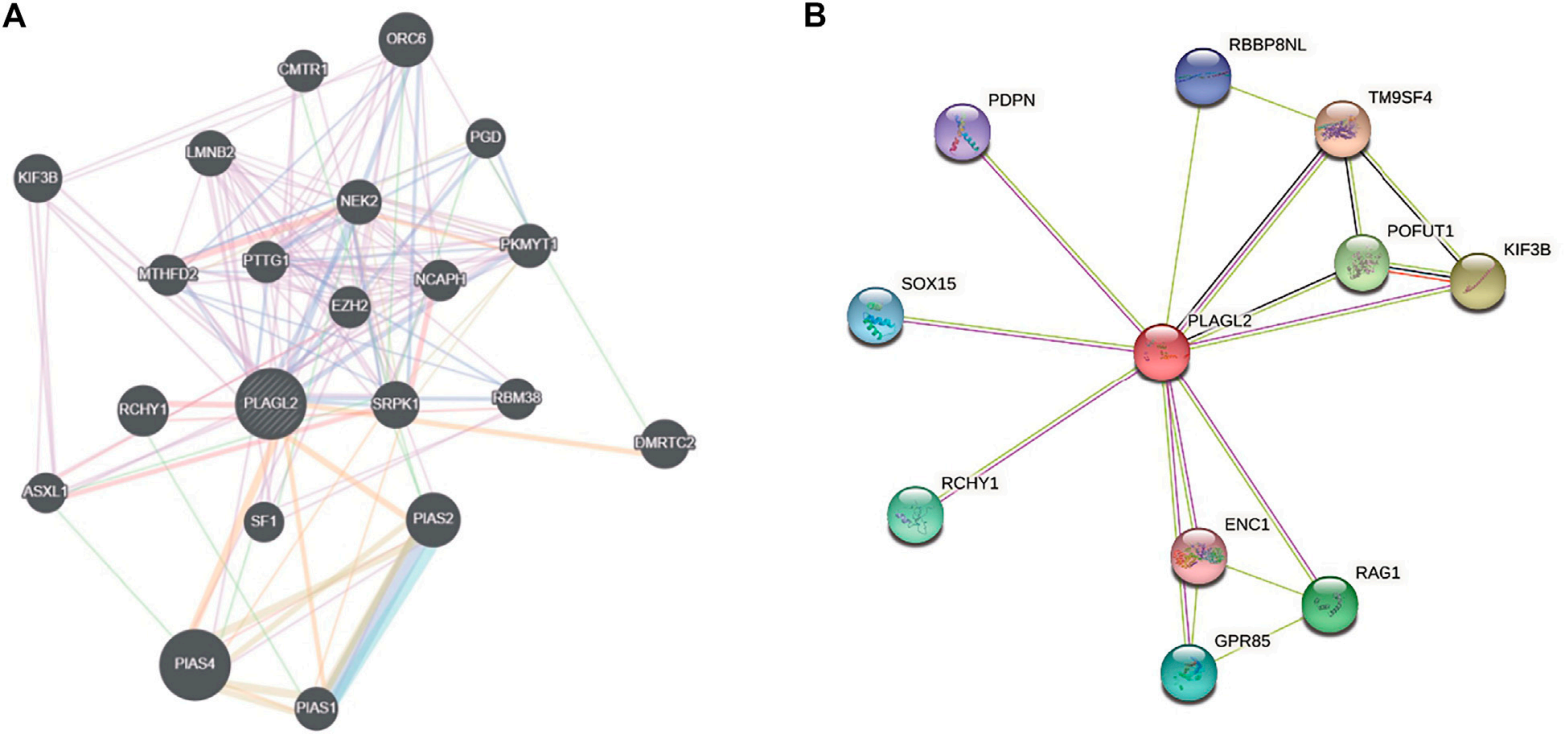
Interaction network of PLAGL2 gene arisen from GeneMANIA and STRING database. **(A)** based on physical and genetic interactions, pathway analysis, co-expression, and localization, GeneMANIA performed the interactions for PLAGL2 linked with each other. **(B)** The protein interaction network against PLAGL2 was conducted in the STRING database.

An aberrant expression of PLAGL2 may be involved in the process of diverse cancer types. Herein, we implemented two web-based network tools, GeneMANIA, and STRING, to investigate the interaction network related to PLAGL2. It is well known that protein mediates a wide variety of cellular functions and biological processes regarding protein interaction and the signal transduction pathway (Pawson and Nash, 2000; Westermarck et al., 2013). GeneMANIA, as an integrated network, focuses on functional prediction and performs an interaction network analysis based on a network-based gene ranking algorithm. Meanwhile, STRING is more inclined to the physical and functional interactions of a gene set. As Figure 7A showed, GeneMANIA supplied that the PPI-associated protein included ORC6, CMTR1, KIF3B, LMNB2, PGD, NEK2, MTHFD2, PTTG1, EZH2, NCAPH, PKMYT1, RCHY1, SRPK1, RBM38, DMRTC2, ASXL1, SF1, PLAS2, PIAS4, and PIAS1. STRING analysis provided a predicted PPI network, which shares interaction with RBBP8NL, PDPN, TM9SF4, SOX15, POFUT1, KIF3B, RCHY1, ENC1, RAG1, and GPR85 (Figure 7B). The PPI network stats are the number of nodes: 11; the number of edges: 17; average node degree: 3.09; local clustering coefficient: 0.893, and PPI enrichment *p* < 0.05. The related parameters predicted that PLAGL2 is involved in the progression and prognosis of cancer.

### Gene Set Enrichment Analysis for PLAGL2 Expression in Glioma

**FIGURE 8:**
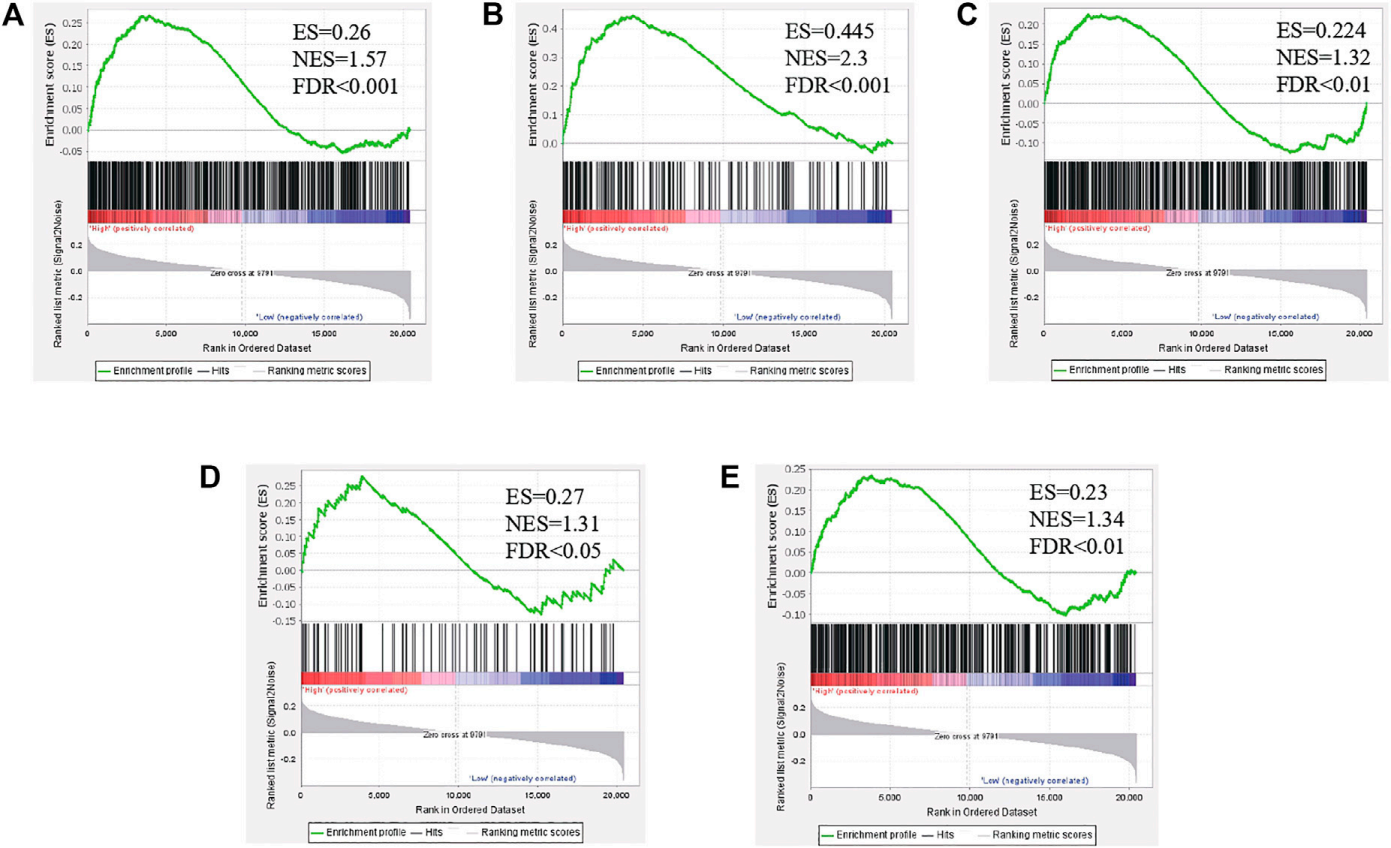
The level of PLAGL2 was positively associated with migration, proliferation, cytoskeleton, and angiogenesis in clinical specimens. **(A–E)** Enrichment plots of gene expression signatures according to PLAGL2 mRNA expression levels by the GSEA of glioma databases GSE4290. HGG samples were divided into high and low PLAGL2 expression groups. Gene signatures of migration, proliferation, cytoskeleton, angiogenesis, and VEGF-VEGFR2 signaling pathway assembly were enriched in high-expression specimens compared to low-expression patients. False discovery rate (FDR) showed the estimated probability that a gene set with a given normalized ES (NES) represented a false-positive finding; FDR<0.05 was a widely accepted cut-off for the identification of biologically significant gene sets.

To investigate the biological characteristics shared by the different PLAGL2 expression levels on proliferation, migration, and F-actin polymerization of gliomas, we divided HGG patients from GSE4290 into PLAGL2-positive and PLAGL2-negative groups and performed GSEA, a robust computational method that determines whether an *a priori* defined set of genes is statistically significant, as well as the concordant differences between both groups. The enrichment plots of GSEA showed that the gene signatures of proliferation were enriched in PLAGL2-positive glioma HGG tissues compared with PLAGL2-negative groups (Figure 8A). In addition, similar enrichment was found related to the gene signatures of migration when the PLAGL2-positive glioma HGG tissues were compared with PLAGL2-negative groups (Figure 8B). Furthermore, the cytoskeleton assembly gene sets were enriched in PLAGL2-positive glioma HGG tissues as compared with PLAGL2-negative groups (Figure 8C). Accordingly, GSEA results further indicated that glioma with various PLAGL2 mRNA expression levels had the distinct status of angiogenesis (Figure 8D). The VEGF-VEGFR2 signaling pathway represented a growth factor with important pro-angiogenic activity, having a mitogenic and anti-apoptotic effect on endothelial cells, increasing the vascular permeability, and promoting angiogenesis (Ferrara et al., 2003). The gene signatures of the VEGFA-VEGFR2 signaling pathway assembly were abundantly activated in patients with a higher expression of PLAGL2 (Figure 8E).

## Discussion

PLAGL2 has been identified as the cause of various cancer tumorigeneses and has oncogenic and tumor suppressor activities in different cancers (Landrette et al., 2005; Zheng et al., 2010; Hanks and Gauss, 2012; Li et al., 2019). High PLAGL2 mRNA levels were correlated with a worse prognosis in several cancers, including colorectal cancer, HCC, gastric cancer, breast cancer, and urothelial bladder carcinoma (Qu et al., 2018; Li et al., 2019; Fan et al., 2021; Hu et al., 2021; Wu et al., 2021). However, few studies reported on the expression of PLAGL2 and its role in the progression and tumor initiation of malignancy in gliomas.

Glioma, the most common malignancy of the CNS, is characterized by rapid proliferation, relentless migration, a dysregulation of the cytoskeleton, and robust angiogenesis. The poor prognosis of gliomas has been associated with the near-universal recurrence of tumors despite aggressive multimodality treatment of maximal surgical resection, radiotherapy, and chemotherapy (Stupp et al., 2005; Omuro and Deangelis, 2013).

PLAGL2 mRNA levels in cancer tissues were analyzed using the TIMER and Gent2 databases. An analysis of PLAGL2 mRNA levels with cancer and normal tissues in the TIMER and Gent2 databases showed that PLAGL2 expression was critically upregulated in most cancers. Similarly, the PLAGL2 expression level was consistent in GBM tissues against various databases. Herein, we attempted to clarify whether there was any aberrant expression of PLAGL2 in glioma and identify the role of PLAGL2 in glioma progression and prognosis.

Our results initially confirmed the PLAGL2 mRNA levels between glioma and non-neoplastic brain tissues in GBM and LGG. We found that upregulated PLAGL2 in human glioma compared with normal brain tissues. The expression of PLAGL2 was elevated as the tumor grade increased. Our findings were identical with previous results, suggesting that PLAGL2 expression changes in different cancers. Aberrant PLAG2 expression was found in the development of the expression of PLAGL2 bladder urothelial carcinoma, AML, gastrointestinal cancers, and glioblastoma (Landrette et al., 2005; Zheng et al., 2010; Liu et al., 2014; Li et al., 2019). Accumulating studies have focused on several fundamental cellular processes of PLAGL2 involving crucial mechanisms in tumorigenesis, which is a potential prognostic biomarker in HGG. Nonetheless, the exact role of PLAGL2 and the underlying mechanism in gliomas continue to remain largely unknown.

A high expression of PLAGL2 was observed in the glioma sample and located in the nuclei of tumor cells based on a cohort of HGG samples examined by IHC. Given these results, the data supported the concept that PLAGL2 was upregulated in HGG. Several studies have already demonstrated that overexpressed PLAGL2 possessed oncogenic potentiality in tumors (Landrette et al., 2005; Xu et al., 2018; Li et al., 2019). Furthermore, PLAGL2 could induce the proliferation of NIH-3T3 cells and participate in the AML progression in cooperation with the CBFB-MYH11 fusion gene that encodes the fusion protein CBFβ-SMMHC (Landrette et al., 2005). A role of PLAGL2, which needed to be further determined in colorectal cancer, was also identified as a suspected candidate of lung adenocarcinoma as patients with low PLAGL2 expression had a better prognosis at the early stage of disease (Yang et al., 2011). Thus, further investigation was implicated in elucidating whether PLAGL2 overexpression could have a vital role in the progression of glioma. By analyzing the correlation between PLAGL2 expression and clinicopathological features in HGG patients, we found that PLAGL2 expression was only associated with tumor grade. Furthermore, multivariate analysis illustrated that PLAGL2 expression was an independent prognostic factor for OS and PFS. Accordingly, the current study showed that patients with high PLAGL2 expression had significantly poor OS and PFS in HGG cohort. Given the mentioned results, the relationship between high PLAGL2 expression and poor prognosis was warranted.

Our results also revealed that PLAGL2 expression was associated with infiltration levels of immune cells in GBM. There was a strong positive association of PLAGL2 with the expression and infiltration of immune cells, including B cells, CD4^+^ T cells, macrophages, neutrophils, and DCs. These findings suggested that PLAGL2 was closely correlated with the infiltration of immune cells in GBM, which has a critical role in modulating tumor immunity. Moreover, we found a correlation between the expression of PLAGL2 and monocyte marker, CD14 and CD115, M1 macrophage markers, CCL5, IL18, COX, M2 macrophage marker, CD163, and VSIG4. This suggested that PLAGL2 regulated the infiltration and activity of TAMs. Meanwhile, PLAGL2 expression was also correlated with the expression of immune cell markers of various subsets of T-helper (Th) cells, including Th1 (TBX2), Th2 (CD14, GATA3, STAT6, and STAT5A), Tfh (BCL6), Th17(STAT3) and Tregs (FOXP3, STAT5B, and TGF-β), which also had a potential influence in regulating the tumor immunity infiltration of T-helper cells for PLAGL2.

So far, the role of PLAGL2-induced oncogenesis was still somewhat unclear. PLAGL2 impeded the differentiation of neural stem cells through WNT/β-catenin signaling (Zheng et al., 2010). NIH3T3 cells overexpressing PLAGL2 can induce tumors in nude mice, displaying the typical markers of neoplastic transformation attributed to the activation of insulin-like growth factor-II mitogenic pathway (Hensen et al., 2002). Conversely, Furukawa *et al.* (Furukawa et al., 2001) found that PLAGL2 involved cell apoptosis in response to iron deficiency and hypoxia activation. Other studies have shown that PLAGL2, as a tumor suppressor gene, could also induce cell cycle block and the apoptosis of human promonocytic U937 cells by regulating the expression of the p73 (Hanks and Gauss, 2012).

PLAGL2 was distinctly expressed in different tumors with tissue specificity. The contrary PLAGL2 function attributed to different modulatory mechanisms and contributing to glioma tumorigenesis needs further investigation. The enrichment plots of GSEA analysis showed that PLAGL2 expression was positively associated with the activation of genes of cell migration, proliferation, actin cytoskeletal, and angiogenesis assembly in clinical specimens. Thus, it suggested that PLAGL2 expression could be associated with the progression and development of glioma. Interestingly, GSEA analysis showed that PLAGL2 expression was positively correlated with the VEGFA-VEGFR2 signaling pathway. To the best of our knowledge, gliomas are highly vascular tumors, which require a longstanding therapeutic approach for the suppression of angiogenesis (Norden et al., 2008). Thus, further work is needed to elucidate the role, and function of PLAGL2 in tumor angiogenesis, which could provide not only evidence-based mechanisms of GBM-acquired resistance to anti-angiogenic treatment but also a proof of concept for translational research by combining anti-angiogenic therapy to effectively eradicate GBM, thus constituting a landmark clinical advance for GBM.

The present study had some limitations. Firstly, multiple databases could cause some bias because of the differences in sample size. In addition, the study only supplied the mRNA level of PLAGL2, regardless of protein or its posttranslational levels. Finally, the molecular mechanism and effect for clinical translational therapy should be further investigated.

In conclusion, this was the first study that evaluated the upregulated expression of PLAGL2 in gliomas compared with normal brain tissue. Additionally, the overexpression of PLAGL2 was correlated with adverse clinicopathological characteristics, such as tumor grade. Our study also demonstrated that PLAGL2 was an independent prognostic indicator for PFS and OS in glioma clinical specimens. Furthermore, the present study also furthered the understanding of the differential mechanisms positively associated with PLAGL2 expression in the progression of gliomas, which may serve as a favorable prognostic marker in the diagnosis and prognostic of gliomas. Yet, the precise mechanism of PLAGL2 action on gliomas angiogenesis needed to be further investigated. We hoped that our findings could have significant implications for diagnosis and therapies designed to treat patients with HGG and prevent HGG.

## Data Availability

The original contributions presented in the study are included in the article/Supplementary Material, further inquiries can be directed to the corresponding authors.
