# Supplementary figures and images for: High Expression of PLAGL2 is Associated With Poor Prognosis in High-Grade Glioma
Source: Front Genet. 2022 Feb 9;12:787746. doi: 10.3389/fgene.2021.787746 (PMC8863765; doi:10.3389/fgene.2021.787746)

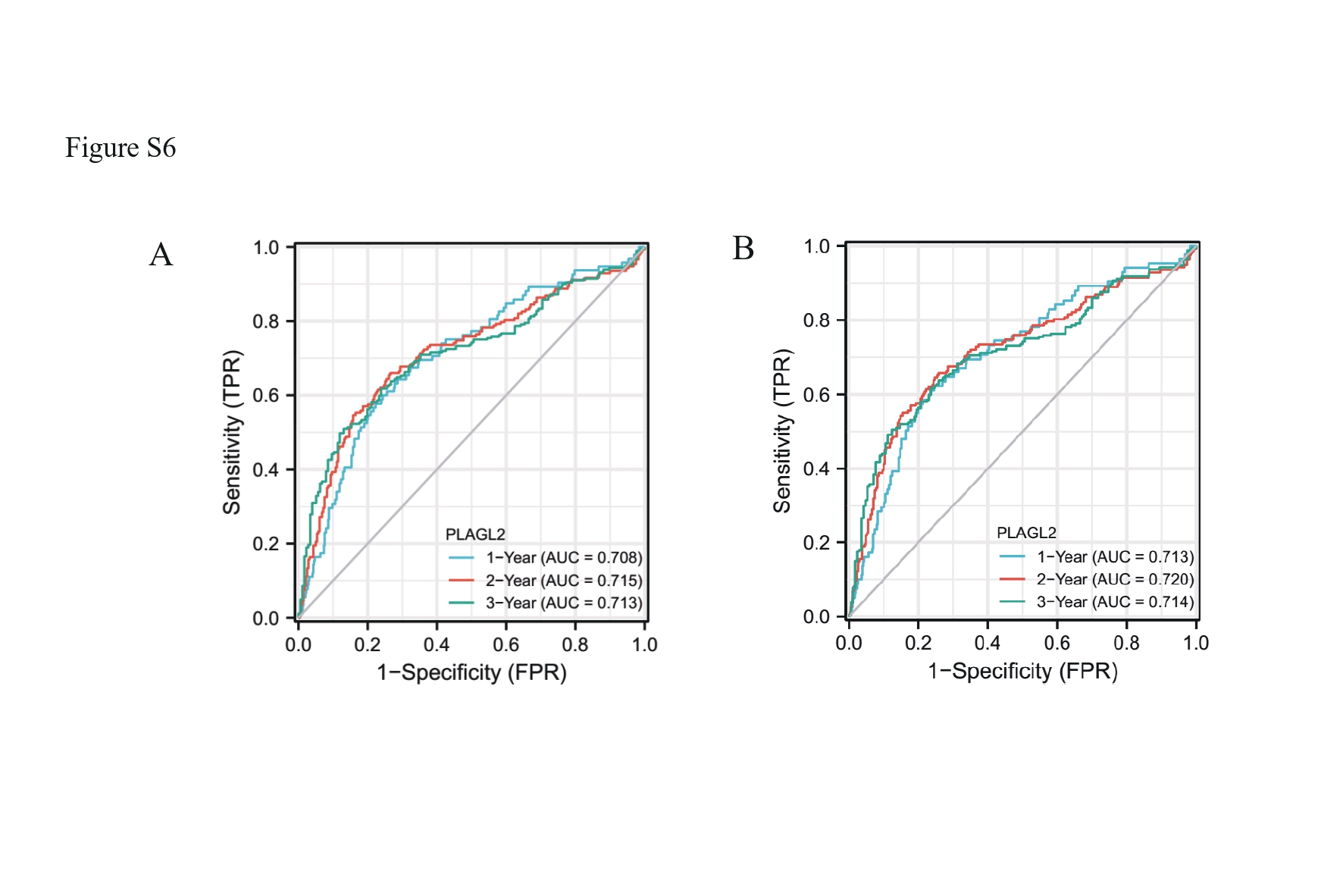

Supplement: Supplementary file 2 [file Image1.JPEG]

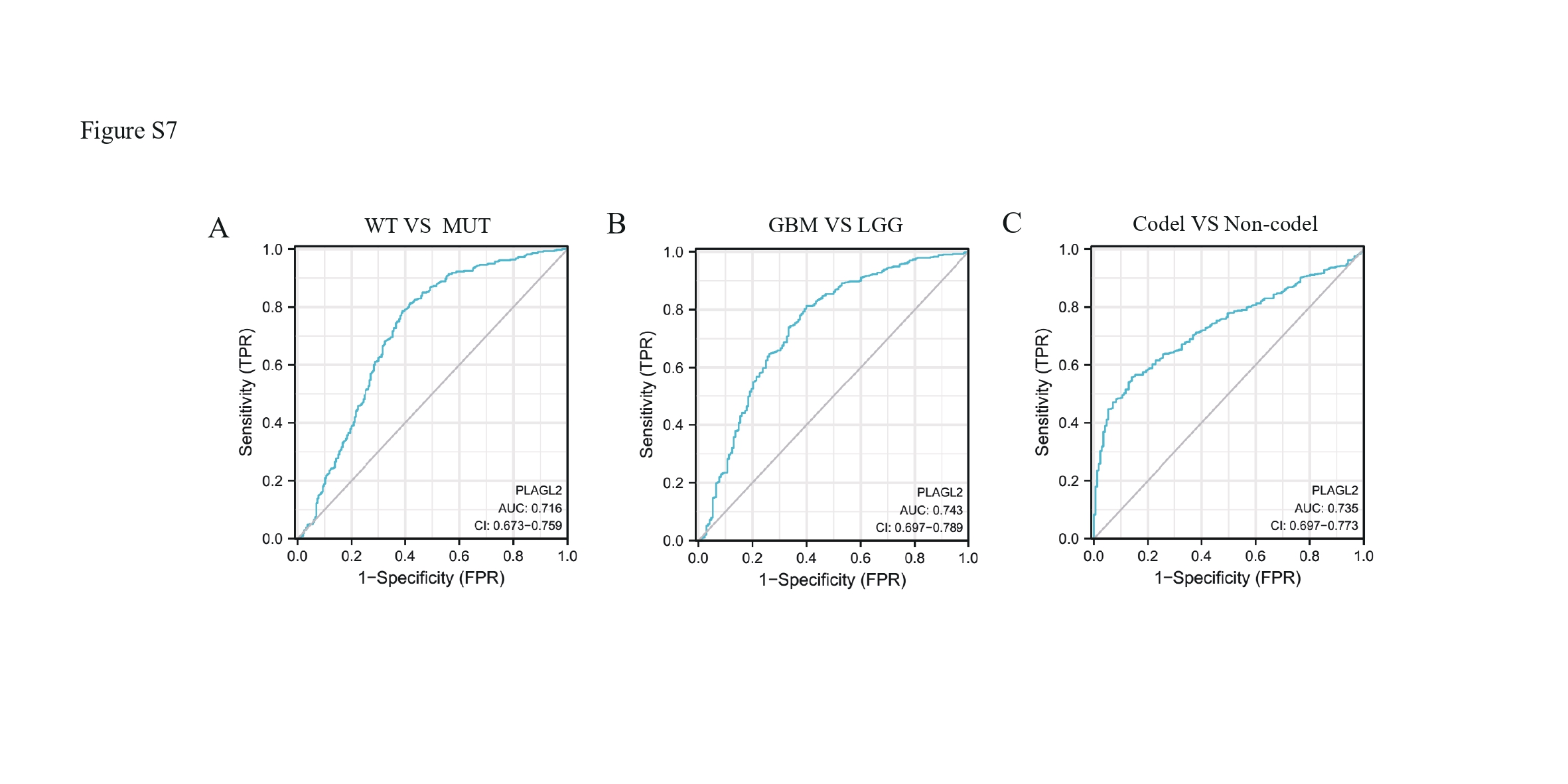

Supplement: Supplementary file 3 [file Image2.JPEG]
